# Integrated Functional, Gene Expression and Genomic Analysis for the Identification of Cancer Targets

**DOI:** 10.1371/journal.pone.0005120

**Published:** 2009-04-09

**Authors:** Elizabeth Iorns, Christopher J. Lord, Anita Grigoriadis, Sarah McDonald, Kerry Fenwick, Alan MacKay, Charles A. Mein, Rachael Natrajan, Kay Savage, Narinder Tamber, Jorge S. Reis-Filho, Nicholas C. Turner, Alan Ashworth

**Affiliations:** 1 The Breakthrough Breast Cancer Research Centre, The Institute of Cancer Research, London, United Kingdom; 2 Genome Centre, Bart's and the London Hospital, Queen Mary's School of Medicine and Dentistry, The Sir John Vane Science Centre, London, United Kingdom; Northwestern University, United States of America

## Abstract

The majority of new drug approvals for cancer are based on existing therapeutic targets. One approach to the identification of novel targets is to perform high-throughput RNA interference (RNAi) cellular viability screens. We describe a novel approach combining RNAi screening in multiple cell lines with gene expression and genomic profiling to identify novel cancer targets. We performed parallel RNAi screens in multiple cancer cell lines to identify genes that are essential for viability in some cell lines but not others, suggesting that these genes constitute key drivers of cellular survival in specific cancer cells. This approach was verified by the identification of *PIK3CA*, silencing of which was selectively lethal to the MCF7 cell line, which harbours an activating oncogenic *PIK3CA* mutation. We combined our functional RNAi approach with gene expression and genomic analysis, allowing the identification of several novel kinases, including *WEE1*, that are essential for viability only in cell lines that have an elevated level of expression of this kinase. Furthermore, we identified a subset of breast tumours that highly express WEE1 suggesting that WEE1 could be a novel therapeutic target in breast cancer. In conclusion, this strategy represents a novel and effective strategy for the identification of functionally important therapeutic targets in cancer.

## Introduction

Central to the design of novel therapeutic strategies for cancer is the identification of genes that are critical to the survival of tumour cells but which are largely redundant in normal cells [1]. Correlating molecular changes with tumourigenesis has provided one route to the identification of potential drug targets and provides the rationale behind efforts to characterise genetic variation and gene expression in tumours. However, the correlative nature of these data means that it is frequently not possible to determine whether the observations are causative or merely an effect of the disease state [2].

RNA interference (RNAi) is a naturally occurring mechanism that regulates gene expression at the post-transcriptional level. In mammalian cells, short-interfering RNAs (siRNAs) mediate the degradation of complementary messenger RNA (mRNA) transcripts in a sequence-dependent fashion [3]. This sequence-specificity of RNAi can be utilised experimentally to silence specific genes by the transfection of siRNAs into mammalian cells. This technology has been expanded into RNAi libraries encompassing reagents that target a wide range of transcripts, allowing the role of multiple genes in a cellular process to be assessed in an unbiased fashion [4], [5]. RNAi screens have been used to identify genes important for cancer cell phenotypes, including cell viability [6], [7].

We demonstrate that RNAi screens can be used to identify genes that are differentially required for viability of cancer cell lines and, as proof of this principle, identify the known oncogene *PIK3CA* as essential for viability in MCF7 cells with an activating *PIK3CA* mutation. We show that combining functional RNAi analysis with gene expression and genomic analysis provides a new strategy for the identification of key drivers of specific cancer cells, which are potential novel drug targets.

## Results

### Parallel RNAi screens to identify kinases essential for cell viability

**Figure 1:**
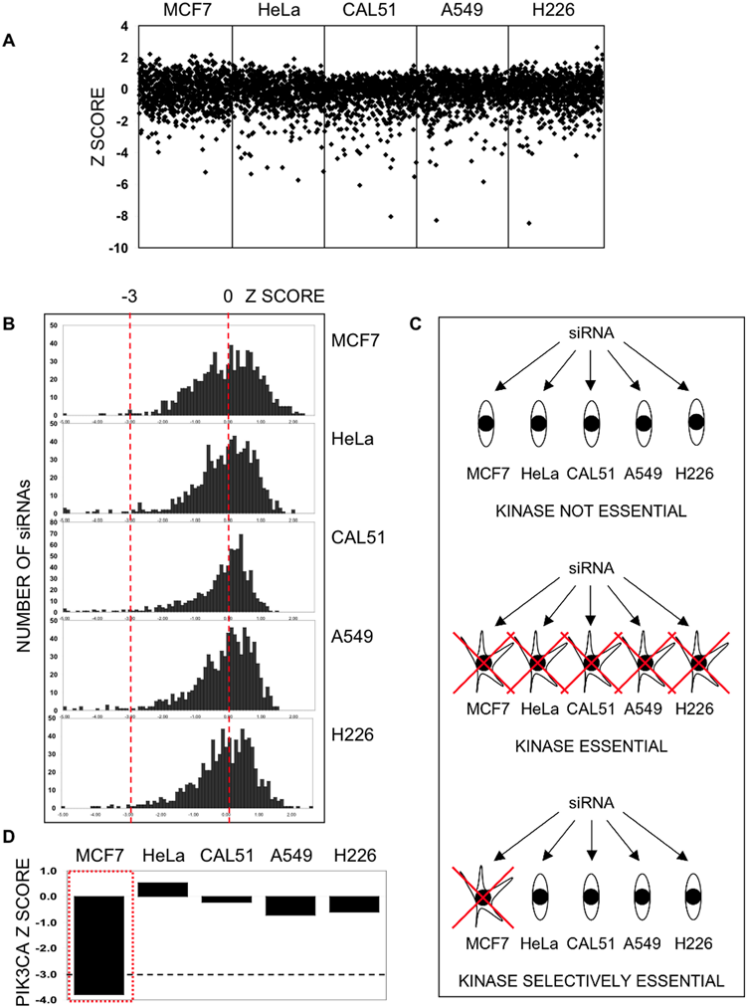
Cell viability screens with a kinase siRNA library. a. Scatter plots of Z scores from cell viability screens carried out in parallel in MCF7, CAL51, HeLa, A549 and NCI-H226 cancer cell lines. Black diamonds – individual siRNA SMARTpools targeting 779 kinase genes per cell line. Z scores≤−3 represent significant loss of viability effects. b. Distribution plots of Z scores from the parallel siRNA screens. Z scores≤−3 represent significant loss of viability effects. c. Kinases can be classified on the basis of the effect of silencing on cell viability across all five cancer cell lines. siRNAs that had no significant effect on cell viability in any of the cell lines studied likely target nonessential kinases (or the siRNA was not functional). siRNAs that cause significant loss of cell viability in all of the cell lines studied likely target kinases that are essential for viability in most tumour types or those that are essential for the viability of both normal and tumour cells. siRNAs that only cause significant lethality in some but not all cell lines likely target kinases that may not be critical for the viability of all cells but represent tumour-specific effects. d. Parallel RNAi screens identify a known oncogene, *PIK3CA*. Cell viability effects of *PIK3CA* targeting are shown in five cell lines. MCF7 cells were selectively sensitive to targeting of *PIK3CA* as demonstrated by a Z score of ≤−3. siRNAs that cause significant lethality in some but not all cell lines are likely to target kinases that are not critical for the viability of all cells but represent tumour-specific effects. In some instances this may be explained by the occurrence of an activated oncogene as is the case with MCF7 cells, which harbour an activating *PIK3CA* mutation.

To functionally identify important genes expressed in cancer cells, we used an RNAi screening approach. Using a diverse range of human cancer cell lines and a short interfering RNA (siRNA) library targeting 779 kinases, we performed five parallel viability screens using MCF7 (ER positive, luminal breast cancer), CAL51 (ER negative, microsatellite unstable breast cancer), A549 (lung cancer), NCI-H226 (lung cancer) and HeLa (cervical cancer) cell lines (Figure 1a and Table S1). We chose to target kinases as these proteins are relatively amenable to pharmacological inhibition and have been shown to be important drivers of many different cancers. In brief, cells were plated in 96 well plates and transfected with siRNA from the library. Here we used a SMARTpool library, where each well of the 96 well-plate contained a pool of four different siRNAs (a SMARTpool) targeting one gene. After seven days continuous culture, cell viability in each well was estimated by use of a luminescent assay measuring cellular ATP levels. In order to compare loss of viability effects in different cell lines, we normalised cell viability data from each cell line to the median of all effects in that cell line, representing each SMARTpool effect as a Z score [8] where Z = 0 represented no effect on viability and Z scores less than −3 represented significant loss of viability effects. The results from the five cell viability screens approximated normal distributions, allowing comparison of the individual siRNA effects across cell lines (Figure 1b).

We reasoned that siRNAs causing significant loss of cell viability (Z≤−3) in all of the cell lines assayed likely represented kinases that are essential for viability in most tumour types or more likely essential for the viability of both normal and tumour cells. Similarly, siRNAs that had no significant effect on viability in any of the cell lines were either not functional or targeted non-essential kinases. Finally, we hypothesised that siRNAs that only caused significant lethality in some but not all cell lines, identified kinases that represent tumour-specific effects potentially identifying new therapeutic targets (Figure 1c). To determine the nature of the effect, siRNAs were classified by comparing Z scores between cell lines (Table 1).

**Table 1:** Results of parallel siRNA screens.

GENE	AC. NO.	MCF7 Z SCORE	HeLa Z SCORE	CAL51 Z SCORE	A549 Z SCORE	H226 Z SCORE	Pearson r	P
ADCK2	NM_052853	**−3.06**	0.74	0.34	−0.59	−1.99	−0.93	0.02
AURKB	NM_004217	−1.42	**−3.65**	**−3.47**	−1.33	−0.93	−0.56	0.32
BUB1B	NM_001211	**−3.03**	**−4.33**	−1.60	−1.96	−1.78	0.06	0.93
CALM3	NM_005184	−1.01	−2.71	−1.88	−1.23	**−3.54**	0.2	0.74
CDC2L2	NM_024011	−1.92	**−4.05**	**−4.36**	**−4.56**	**−3.62**	0.5	0.39
CDK9	NM_001261	0.91	−1.80	−1.35	**−3.76**	−0.18	−0.46	0.44
CHKA	NM_001277	−0.27	−0.38	−0.59	−2.00	**−3.08**	−0.18	0.77
CIT	NM_007174	−0.93	**−4.09**	−1.21	−1.24	**−3.54**	0.5	0.39
CNKSR1	NM_006314	**−3.36**	**−4.19**	**−3.01**	**−4.78**	**−3.92**	0.65	0.23
COPB2	NM_004766	**−3.96**	**−5.34**	**−3.81**	**−8.26**	**−8.44**	0.1	0.87
CSNK1G1	NM_022048	−1.06	−2.81	**−4.12**	−1.98	−1.06	0.04	0.94
DGKE	NM_003647	−0.42	0.30	−2.50	−1.40	**−4.05**	0.34	0.58
EXOSC10	NM_002685	−2.06	−2.18	**−3.10**	−1.04	−2.09	−0.71	0.18
FGFR3	NM_000142	−0.60	−0.39	−0.28	**−3.43**	−0.40	0.64	0.25
GALK1	NM_000154	−1.13	−1.50	**−3.88**	−0.92	−1.03	0.18	0.77
GALK2	NM_002044	−0.73	−0.38	−0.86	**−3.30**	−0.40	0.21	0.73
GUCY2D	NM_000180	−1.46	−0.61	**−6.05**	−2.10	−0.76	−0.36	0.55
GUK1	NM_000858	−1.33	**−4.94**	**−4.04**	−2.34	0.41	0.26	0.68
LMTK3	XM_055866	−2.24	−2.73	**−3.60**	−2.19	−0.07	0.01	0.99
MASTL	NM_032844	−0.35	**−4.93**	0.24	−1.15	−1.00	0.13	0.84
MYLK2	NM_033118	−2.40	−1.14	−0.55	**−3.15**	−1.56	0.35	0.57
NAGK	NM_017567	−1.25	−1.26	−0.50	**−4.53**	**−3.08**	−0.97	0.01
NME3	NM_002513	−0.71	−1.71	**−3.20**	−1.07	−0.19	0.83	0.08
PANK4	NM_018216	−1.27	**−3.24**	−1.89	−2.79	−2.41	−0.02	0.98
PFKFB1	NM_002625	−0.95	−2.41	**−4.13**	−0.67	−0.75	0.8	0.1
PIK3C2A	NM_002645	−2.99	−1.13	**−3.30**	−0.69	−1.99	−0.53	0.36
PIK3CA	NM_006218	**−3.80**	0.54	−0.22	−0.73	−0.60	−0.94	0.02
PKN3	NM_013355	−0.28	−0.98	**−4.95**	0.11	−0.13	−0.7	0.19
PLK1	NM_005030	**−5.23**	**−5.72**	**−8.03**	**−5.83**	**−4.18**	−0.57	0.32
PMVK	NM_006556	−0.84	−0.59	−0.49	**−3.74**	−2.28	0.72	0.17
PRKAG3	NM_017431	−2.04	**−3.11**	**−4.43**	**−3.27**	−0.09	0.2	0.74
RPS6KA2	NM_021135	**−3.14**	−2.66	−1.90	**−3.74**	−2.89	0.95	0.01
SYK	NM_003177	−1.77	−2.09	−1.05	**−4.06**	−0.40	0.16	0.8
TLR6	NM_006068	−0.71	−0.77	−0.04	**−3.46**	−0.19	−0.97	0
TTK	NM_003318	−2.01	**−3.66**	**−5.51**	−2.06	−2.22	−0.45	0.44
WEE1	NM_003390	−1.00	**−5.18**	**−4.61**	0.08	−0.08	−0.88	0.05

siRNAs causing loss of viability (where Z≤−3) are shown for five cell lines. Z scores of ≤−3 are shown in bold. Pearson correlation coefficient for correlation with gene expression, with P value.

### Identification of *PIK3CA* provides proof of principle for the approach

Our initial analysis indicated that *PIK3CA* silencing was likely to represent a cell line specific effect. Silencing of *PIK3CA* was selectively lethal to MCF7 cells (Z score of −3.80) but not HeLa, CAL51, A549 nor H226 (Figure 1d and Table 1). MCF7 cells are known to harbour an activating *PIK3CA* mutation (E545K) on which these cells are dependent for survival [9], [10]. Furthermore, amplifications and gain-of-function mutations of *PIK3CA* have been associated with ovarian cancer [11], cervical cancer [12] and breast cancer [10]. The dependence of MCF7 cells upon a *PIK3CA* activating mutation, may be an oncogene addiction effect which may be exploited therapeutically [13]. In cases of gene addiction, tumour cells become physiologically dependent upon the continued function of activated or overexpressed oncogenes which are therefore obvious candidate therapeutic targets. For example, the efficacy of imatinib (Gleevec) in the treatment of leukaemias bearing the BCR-ABL fusion [14] provides one clinical example of oncogene addiction and how it may be exploited therapeutically. The identification of *PIK3CA* validated our approach to identify kinases that are essential for tumour cell survival.

### Correlation of cell viability with gene expression

**Figure 2:**
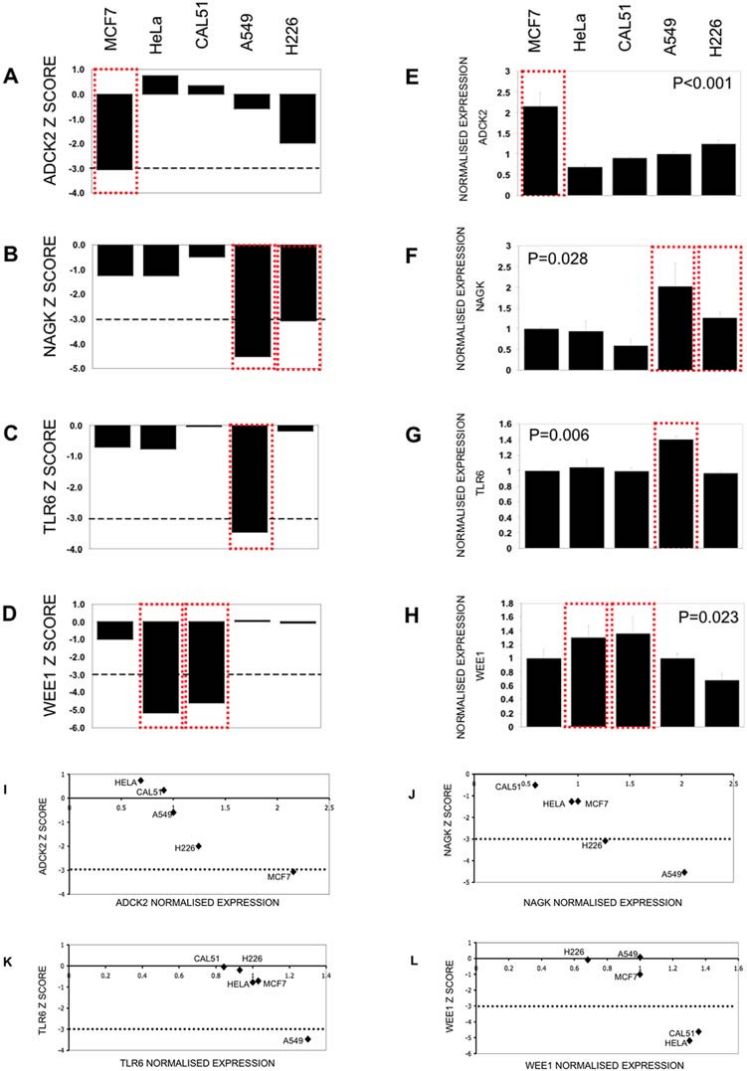
Correlation between siRNA loss of viability and gene expression. a–d. Z scores from the siRNA screens, Z scores≤−3 are highlighted with a red dotted box. e–h. Normalised expression levels calculated from Illumina expression profiling. High expression correlated with sensitivity to siRNA, highlighted with a red dotted box. Error bars represent the standard error of the mean (SEM). The significance of differences in genes expression between cell lines was assessed by one-way ANOVA and p value displayed for each cell lines. i–l. comparison of Z values with normalised expression levels. The dashed line represents the Z = −3 threshold for significant loss of viability effects (p<0.0015). For the four genes shown, an elevated level of expression is consistent with loss of viability after siRNA transfection. See Table 1 for Pearson correlation of Z vs expression.

Although cell-specific gene effects identified in the RNAi screen may be because of activating mutations, such as in *PIK3CA*, it is likely that others could arise because of the acquisition of an increased level of gene expression. To investigate this, we performed genome-wide gene expression profiling on the cell line panel using human-6 v2 Illumina BeadChips [15] and compared this to the RNAi screen data (Table S2). Expression profiling was performed in triplicate and kinases with significant differences in gene expression between cell lines identified by analysis of variance. For genes where at least one siRNA significantly decreased cell viability (Table1), we examined the correlation between cellular viability following siRNA transfection and gene expression. This analysis identified four genes where cellular viability inversely correlated with gene expression; *ADCK2*, *NAGK*, *TLR6* and *WEE1* (Figure 2 and Table 1). Each of these correlations suggested that elevated expression of the gene in question may be essential for tumour survival and may therefore represent a novel therapeutic target.

The Toll-like receptor 6, (TLR6) is known to activate nuclear factor kappa-B signalling, a candidate therapeutic target in cancer [16], and activation of the TLR pathway has recently been suggested to have a role in tumourigenesis [17]. The function of *ADCK2* (aarF domain containing kinase 2) is less well established. NAGK (N-acetylglucosamine kinase) converts endogenous N-acetylglucosamine (GlcNAc), a major component of complex carbohydrates, from lysosomal degradation or nutritional sources into GlcNAc 6-phosphate, as part of a catalytic salvage pathway. The function of WEE1 is discussed below.

### Correlation of gene expression with genomic analysis

**Figure 3:**
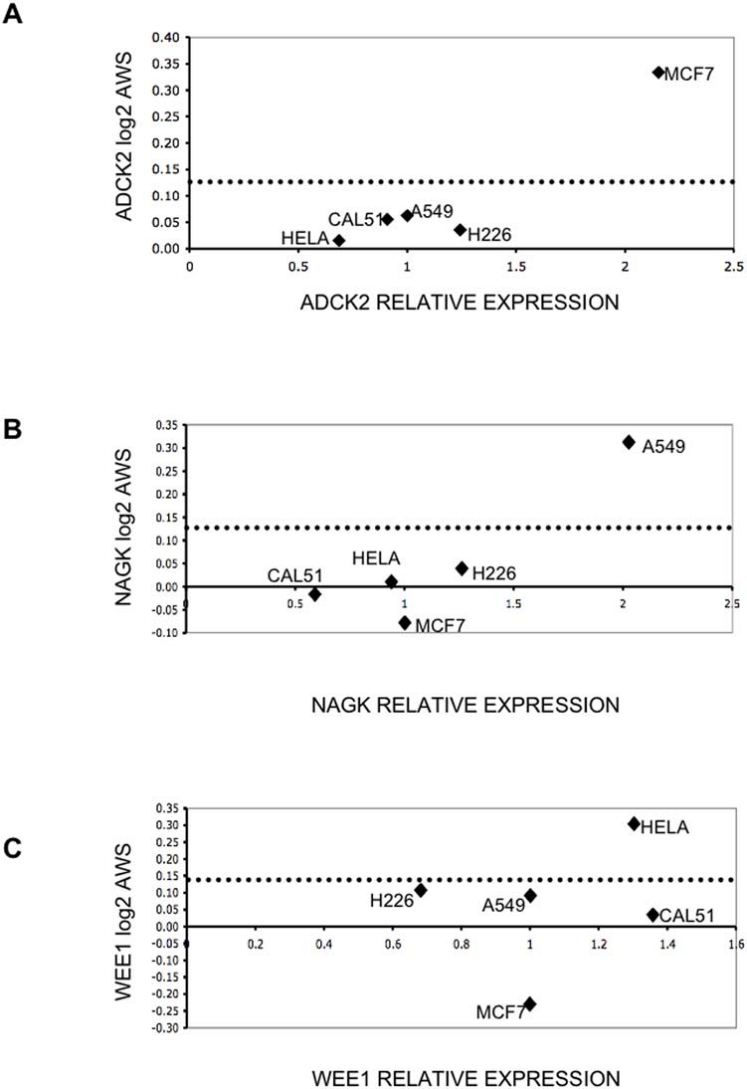
Correlation between gene copy number and gene expression. Scatter plots illustrating the relationship between gene copy number and gene expression. Vertical dashed lines represent the threshold for copy number gains (aws ratios>0.12).

We examined whether the relatively elevated expression of the four genes identified in our RNAi screen could be explained by changes in gene copy number (i.e. copy number gains and/ or gene amplification). Gene copy number was examined using microarray-based comparative genomic hybridisation (aCGH) analysis and overlayed on RNAi and gene expression data (Figure 3, Figure S1 and Table S3). This combined analysis revealed that for some cell lines, elevated expression of *ADCK2*, *NAGK* or *WEE1* was associated with an increase in gene copy number, potentially identifying a cause of elevated expression. MCF7 cells were highly sensitive to *ADCK2* siRNA and this was mirrored by upregulation of the transcript and increase in gene copy number at *ADCK2*. Similarly, an increase in gene copy number was consistent with elevated expression and siRNA sensitivity for *NAGK* in A549 cells. Finally, the sensitivity of HeLa cells to *WEE1* siRNA was consistent with upregulation of this gene and genomic gain (Figure 3 and Figure S1).

### Further characterisation of WEE1

**Figure 4:**
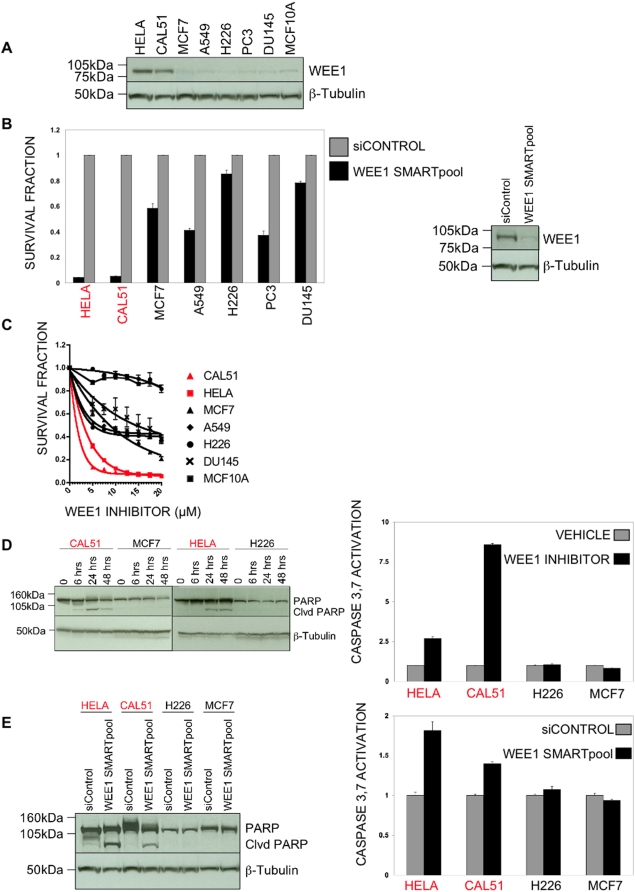
WEE1 expression correlates with sensitivity to WEE1 inhibition. a. Western blot analysis of lysates prepared from HeLa, CAL51, MCF7, A549, NCI-H226, PC3, DU145 and MCF10A cells. An antibody recognising WEE1 was used with β-tubulin as a loading control. WEE1 expression is significantly increased in HeLa and CAL51 cells compared to MCF7, A549, NCI-H226, PC3, DU145 and MCF10A cells. b. Left panel: Cell viability assay in cells transfected with WEE1 ONTARGETplus SMARTpool, or ONTARGETplus siControl. WEE1 silencing was selectively lethal to WEE1 overexpressing HeLa and CAL51 cells. Error bars represent the SEM from triplicate transfections. Right panel: Western blot analysis of lysates prepared from CAL51 cells transfected with WEE1 ONTARGETplus SMARTpool or ONTARGETplus siControl. An antibody recognising WEE1 was used with β-tubulin as a loading control. WEE1 ONTARGETplus SMARTpool significantly reduced WEE1 protein expression compared to siControl transfected cells. c. Cell viability assay in cells treated with WEE1 inhibitor. WEE1 inhibition was selectively lethal to WEE1 overexpressing HeLa and CAL51 cells. Error bars represent the SEM from triplicate cell treatments. d. Left hand panel: Western blot analysis of lysates prepared from cells treated with 5 µM WEE1 inhibitor for 0, 6, 24 and 48 hours. An antibody recognising PARP was used with β-tubulin as a loading control. After 24 hours WEE1 inhibition induced PARP cleavage (Clvd PARP) in WEE1 overexpressing HeLa and CAL51 cells but did not induce PARP cleavage in MCF7 and NCI-H226 cells which express WEE1 at normal levels. Right hand panel: Caspase 3,7 activity in cells treated with 5 µM WEE1 inhibitor for 24 hours. WEE1 inhibition induced caspase 3,7 activation in WEE1 overexpressing HeLa and CAL51 cells but did not induce caspase 3,7 activation in MCF7 and NCI-H226 cells which express WEE1 at normal levels. Error bars represent the SEM from triplicate cell treatments. e. Left hand panel: Western blot analysis of lysates prepared from cells transfected with WEE1 ONTARGETplus SMARTpool or ONTARGETplus siControl. An antibody recognising PARP was used with β-tubulin as a loading control. Silencing of WEE1 induced PARP cleavage in WEE1 overexpressing HeLa and CAL51 cells but did not induce PARP cleavage in MCF7 and NCI-H226 cells which express WEE1 at normal levels. Right hand panel: Caspase 3,7 activity in cells transfected with WEE1 ONTARGETplus SMARTpool or ONTARGETplus siControl. Silencing of WEE1 induced caspase 3,7 activation in WEE1 overexpressing HeLa and CAL51 cells but did not induce caspase 3,7 activation in MCF7 and NCI-H226 cells which express WEE1 at normal levels. Error bars represent the SEM from triplicate transfections.

WEE1 has a well-defined role in cell cycle checkpoint control, with WEE1 activity limiting the pro-mitotic effects of CDC2 (aka CDK1) [18]. Accordingly, loss of WEE1 kinase activity and its destruction is a requirement for entry into mitosis [19], suggesting that WEE1 activity may actually limit tumour cell growth. Given that our RNAi data suggested that WEE1 is, in some contexts, critical for the viability of cancer cells that overexpress it, we investigated the role of this kinase further. We first confirmed that WEE1 protein was overexpressed in HeLa and CAL51 cells supporting our RNA analysis (Figure 4a). To confirm the correlation of sensitivity to siRNA and WEE1 expression, WEE1 was silenced by an independent pool of siRNAs designed to reduce off-target effects (Wee1 ONTARGETplus). CAL51 and HeLa cell lines were significantly more sensitive to silencing of WEE1 than cell lines that do not overexpress WEE1 (Figure 4b and Figure S2). Small molecules have been developed to inhibit WEE1 on the basis that inhibition of this kinase may lead to abrogation of the G_2_/M checkpoint. Many cancer cells exhibit a defective G_1_ checkpoint resulting in a dependence on the G_2_/M checkpoint during cell replication and, as such, inhibition of the G_2_/M checkpoint may be lethal in this context [20]. We used a small molecule WEE1 inhibitor (PHCD [21]) to confirm the selective sensitivity of CAL51 and HeLa cell lines to WEE1 inhibition (Figure 4c). We also examined additional cell lines for WEE1 expression and sensitivity to WEE1 targeting, with prostate carcinoma cell lines PC3 and DU145, and the non-tumourigenic breast epithelial cell line MCF10A. None of these cell lines expressed high levels of WEE1 (Figure 4a), and, as expected, none was sensitive to WEE1 targeting (Figure 4b and 4c).

Taken together, our results provide evidence to suggest that cell lines displaying higher levels of WEE1 expression are sensitive to WEE1 inhibition. To investigate the mechanism of sensitivity in these cell lines, we examined levels of apoptosis following WEE1 inhibition. Chemical inhibition of WEE1 caused apoptosis only in cell lines with higher levels of WEE1 expression (Figure 4d), an observation also confirmed by the use of WEE1 siRNA (Figure 4e).

### Clinical significance of WEE1

**Figure 5:**
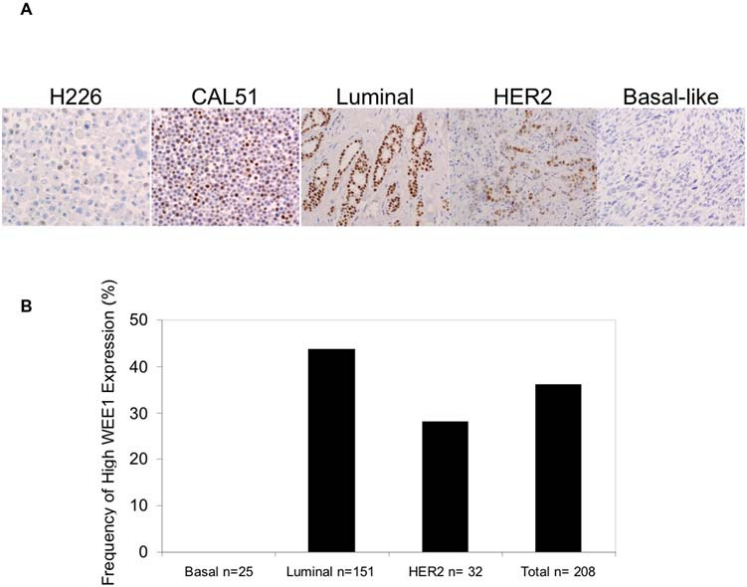
WEE1 is overexpressed in a subset of tumours. a. WEE1 immunohistochemical staining in formalin-fixed, paraffin-embedded breast cancer cell lines and invasive breast cancers. Note the low levels of WEE1 expression in H226 cells and a basal-like breast cancer and the high levels of WEE1 expression in CAL51 cells and luminal and HER2 breast cancers. (Harris Haematoxylin/DAB staining; original magnification ×200). b. High levels of WEE1 expression are preferentially expressed in luminal breast cancers. Cases were scored according to the Allred scoring system [36] as described in the materials and methods. For each tumour type the percentage of tumours with high WEE1 expression is shown. High WEE1 expression showed a statistically significant direct correlation with expression of oestrogen and progesterone receptors and cyclin D1, and a significant inverse correlation with tumour size, histological grade and expression of epidermal growth factor receptor (EGFR), cytokeratin (Ck) 14, Ck 5/6, Ck 17, MIB-1 labelling index and caveolins 1 and 2. No correlations between WEE1 immunohistochemical expression and presence of lympho-vascular invasion, lymph node metastasis, HER2 expression or gene amplification, p53 expression, and *CCND1* and *MYC* gene amplification was found [24], [37] (data not shown). All cases were classified into luminal, HER2 and basal-like groups according to the immunohistochemical panel described by Nielsen et al. [26].

Our data suggest that WEE1 overexpression may be essential for tumour cell viability. Therefore, we interrogated the expression of WEE1 in publicly available datasets that detail the expression profiles of human breast tumour cell lines and tumours [22], [23]. This analysis demonstrated that WEE1 expression correlates with *WEE1* gene copy number (Spearman p = 0.039) and shows a trend (t test p = 0.06, Mann-Whitney Test p = 0.07) for higher expression in cell lines with a luminal phenotype (data not shown). On the basis of these data, we examined WEE1 expression by immunohistochemistry (IHC). Expression of WEE1 assessed by IHC on formalin-fixed, paraffin-embedded cell line pellets correlated with expression measured by western blotting, providing evidence of specificity of the WEE1 antibody (Figure 5a). We then examined a well-annotated series of breast tumours [24], [25] and found that 35% exhibited levels of WEE1 expression similar to those of CAL51 cells, which are sensitive to WEE1 inhibition (Figure 5b). Furthermore, high levels of WEE1 expression were preferentially found in breast cancers with a luminal phenotype, as defined by Nielsen et al. [26], consistent with the analysis of breast cancer cell lines. In the context of our previous data implicating WEE1 as a cancer target, these immunohistochemical data suggest that the use of WEE1 inhibitors may be appropriate in a significant subset of breast cancer patients.

## Discussion

The majority of new drug approvals for cancer treatment are based on existing targets. Rather than reflecting an absence of targets, this is perhaps indicative of the cost and time involved in identifying novel therapeutic approaches. Parallel RNAi screening may allow a simple, high-throughput, approach to the functional identification of targets, and others have also used parallel RNAi screening to identify potential drivers of tumourigenesis and candidate targets [27]. Our identification of *PIK3CA*, a known oncogene and therapeutic target, using parallel RNAi screens provides strong circumstantial evidence for this approach. Furthermore, improvements in RNAi technology may make parallel RNAi screening a much simpler and cost-effective process [28], [29]. Parallel RNAi screens, when combined with companion approaches, such as expression profiling, genomic profiling and high throughput histopathological and immunohistochemical analysis have the potential to identify potential targets that are worthy of further investigation. In the case of WEE1, a combination of RNAi screening, transcript profiling, genomic profiling and histological analysis has led to the identification of a patient subset (luminal breast cancer), where inhibition of this kinase could be explored as a potential therapeutic strategy. Incorporating this approach into the conventional drug target identification process has the potential to streamline the development of new therapies.

## Materials and Methods

### Cell lines, compounds, plasmids and siRNA

MCF7, CAL51, HeLa, A549, NCI-H226, PC3, DU145 and MCF10A cells were obtained from ATCC (USA) and maintained according to the supplier's instructions. WEE1 inhibitor (681637) was obtained from Calbiochem (UK). MCF7 and HeLa cells were transfected with SMARTpool siRNAs using Dharmafect 3 transfection reagent; A549 and NCI-H226 cells were transfected with SMARTpool siRNAs using Dharmafect 1 transfection reagent according to manufacturer's instructions (Dharmacon). CAL51 cells were transfected with SMARTpool siRNAs using Oligofectamine transfection reagent according to manufacturer's instructions (Invitrogen). DU145 cells were transfected with SMARTpool siRNAs using Lipofectamine 2000 transfection reagent according to manufacturer's instructions (Invitrogen). The kinase siRNA library (siARRAY – targeting 779 known and putative human protein kinase genes) was obtained in ten 96 well plates from Dharmacon (USA). Each well in this library contained a SMARTpool of four distinct siRNA species targeting different sequences of the target transcript. Each plate was supplemented with siCONTROL (ten wells, Dharmacon (USA)). The WEE1 ONTARGETplus SMARTpool and ONTARGETplus siControl were obtained from Dharmacon (USA).

### Antibodies

Antibodies targeting the following epitopes were used: WEE1 (4936, Cell Signaling, UK), PARP (9542, Cell Signaling, UK) and β-tubulin (T4026, Sigma, UK). All secondary antibodies used for western blot analysis were HRP conjugated.

### siRNA screen method

Cells plated in 96 well plates were transfected 24 hours later with siRNA (final concentration 100 nM), as per manufacturer's instructions. Each siRNA plate was supplemented with 10 wells of siControl. Twenty four hours following transfection, cells were trypsinised and divided into three identical replica plates. Media was replenished after 48 hours and 96 hours, and cell viability was assessed after seven days using CellTiter Glo Luminescent Cell Viability Assay (Promega, USA) as per manufacturer's instructions. Data from each cell line was processed as follows: the luminescence reading for each well on a plate was log_2_ transformed and expressed relative to the median luminescence value of all wells on the same plate (plate centering). This data was then normalised according to the median of the entire screen data, using the median absolute deviation (MAD) to estimate the true variation within each screen [30]. This normalisation represented the effect of each SMARTpool in each cell line as a Z score [30] and allowed the effects of each SMARTpool on viability to be compared across the cell line panel. A Z score≤−3 was taken as the significance threshold for reduced cell viability, representing three MADs from the median and approximating to three standard deviations.

### Transcript profiling

RNA was extracted from cell lines with Trizol and phenol/chloroform extraction followed by isopropanol precipitation. For reach cell line, triplicate extractions and profiles were performed. Biotin-labeled cRNA was produced by means of a linear amplification kit (IL1791; Ambion, Austin, TX, http://www.ambion.com) using 250 ng of quality-checked total RNA as input. Chip hybridisations, washing, Cy3-streptavidin (Amersham Biosciences) staining, and scanning were performed on an Illumina BeadStation 500 (San Diego, http://www.illumina.com) platform using reagents and following protocols supplied by the manufacturer. cRNA samples were hybridised on Illumina human-6 v2 BeadChips, covering approximately 47,000 RefSeq transcripts. The random distribution of large populations of oligonucleotide-coated beads across the available positions within the human-6 v2 chip enables, on average, 30 intensity measurements per RefSeq, yielding quantitative assessments of gene expression [15]. All basic expression data analysis was carried out using the manufacturer's software BeadStudio 3.1. Illumina expression profiles were performed in triplicate, the raw data were then variance-stabilizing transformed and robust spline normalised using the lumi package in the Bioconductor software [31], [32]. Expression values for each sample were median scaled and the mean expression value was established over the three replicates. Genes with significant difference in expression between cell lines were identified by one-way analysis of variance (ANOVA). This transcript profiling data is now publicly available (ArrayExpress accession: E-TABM-610).

### Correlation of siRNA Z score with gene expression

The correlation between siRNA Z score and normalised gene expression was examined for genes where siRNA caused significant loss of viability (Z<−3). Z score was compared to normalised gene expression using Pearson correlation coefficient. A gene was taken as being significantly correlated if the Pearson correlation coefficient was significantly different to the null hypothesis, the correlation was inverse, and the variation in gene expression between cells lines were significantly different as assessed by one-way ANOVA.

### Array CGH analysis method

Genomic DNA was extracted from cell lines using the QIAamp DNA Blood Mini Kit (51104, Qiagen), according to manufacturer's instructions. Microarray-based CGH analysis was performed on an in-house 32K tiling path BAC array platform as previously described [33], [34]. For copy number correlations, the average of adaptive weight smoothed (AWS) ratios of BACs containing the gene of interest were used for copy number correlations, and copy number assigned as previously described [35]. Briefly, AWS smoothed log2 ratio values <−0.12 were categorised as losses, those >0.12 as gains, and those in between as unchanged. Amplifications were defined as smoothed log2 ratio values >0.4 [35]. Data processing and analysis were carried out in R 2.0.1 (http://www.r-project.org/) and BioConductor 1.5 (http://www.bioconductor.org/), making extensive use of modified versions of the packages aCGH, marray and aws in particular.

### Cell viability assay to measure WEE1 siRNA sensitivity

Cells plated in 96 well plates were transfected 24 hours later with WEE1 ONTARGETplus SMARTpool or ONTARGETplus siControl (final concentration 100 nM), as per manufacturer's instructions. Twenty four hours following transfection, cells were trypsinised and divided into three identical replica plates. Media was replenished after 48 hours and 96 hours, and cell viability was assessed after seven days using CellTiter Glo Luminescent Cell Viability Assay (Promega, USA) and expressed relative to mean luminescence in the wells transfected with siControl.

### Cell viability assay to measure drug sensitivity

Cells were plated in 96 well plates and exposed to various doses of WEE1 inhibitor (Calbiochem, Cat. No. 681637, 4-(2-Phenyl)-9-hydroxypyrrolo[3,4-c]carbazole-1,3-(2H,6H)-dione (PHCD)) [21]. Cell viability was assessed by CellTiter Glo Luminescent Cell Viability Assay (Promega, USA) 48 hours later and surviving fraction for each dose of drug assessed by dividing the luminescence value of drug treated by the luminescence value of vehicle.

### Western blots

Protein lysates were prepared using RIPA lysis buffer (50 nM Tris pH 8.0, 150 mM NaCl, 0.1% SDS, 0.1% DOC, 1% TritonX-100, 50 mM NaF, 1 mM Na_3_VO_4_ and protease inhibitors). 100 µg of total cell lysate was loaded onto prefabricated 4–12% Bis-Tris gels (Invitrogen), with full range rainbow molecular weight marker (GE Healthcare, UK) as a size reference, and resolved by SDS-PAGE electrophoresis. Proteins were transferred to nitrocellulose membrane (Bio-rad, USA), blocked and probed with primary antibody diluted 1 in 1000 in 1×TBS-T with 5% BSA overnight at 4°C. Secondary antibodies were diluted 1 in 5000 in 1×TBS-T with 5% skim milk and incubated for one hour at room temperature. Protein bands were visualised using ECL (GE Healthcare, UK) and MR or XAR film (Kodak).

### Validation of gene silencing by siRNA

Validation of RNAi gene silencing was determined by western blotting. Cells were transfected with WEE1 ONTARGETplus SMARTpool or ONTARGETplus siControl, and protein lysates were made 48 hours later and western blotted for WEE1 expression with β-tubulin as a loading control.

### PARP cleavage western blotting

Cells were transfected with WEE1 ONTARGETplus SMARTpool or ONTARGETplus siControl, and total cell lysates were made 48 hours later and western blotted for PARP with β-tubulin as a loading control. Cells were treated with 5 µM Wee1 inhibitor for 0, 6, 24 and 48 hours. Total cell lysates were made at the time points and western blotted for PARP with β-tubulin as a loading control.

### Caspase 3,7 activation assay

Cells were transfected with WEE1 ONTARGETplus SMARTpool or ONTARGETplus siControl, and caspase 3,7 activation was measured 48 hours later using Caspase-Glo 3/7 Assay (G8091, Promega) and expressed relative to mean luminescence in the wells transfected with siControl. Cells were treated with 5 µM Wee1 inhibitor and caspase 3,7 activation was measured 24 hours later using Caspase-Glo 3/7 Assay (G8091, Promega) and expressed relative to mean luminescence in the wells treated with vehicle.

### WEE1 immunohistochemical staining

Immunohistochemistry for WEE1 was performed with a rabbit polyclonal antibody (Cell Signalling; 4936) at a dilution of 1/20 and developed with the dual Envision kit (Dako®, Glostrup, Denmark). Details of this cohort of patients are described elsewhere [24], [25]. Antigen retrieval was performed at 98°C for 30 minutes in citrate buffer pH 6 (Labvision) in the Labvision pre-treatment module. WEE1 immunohistochemical distribution on tissue microarray sections was analysed by two of the authors (JR-F and KS) on a multi-headed microscope. Only nuclear reactivity was considered specific. Cases were scored according to the Allred scoring system [36] and a cut off of >5 (median score in the series) was adopted. The analysis was performed blinded to the results of other immunohistochemical markers and patients' outcome. All cases were classified into luminal, HER2 and basal-like groups according to the immunohistochemical panel described by Nielsen et al. [26].

## Supporting Information

Table S1 Title of dataset: Z scores for 779 siRNA SMARTpools in five cancer cell lines Description of dataset: The sensitivity of five cancer cell lines to siRNA SMARTpools is shown. Analysis was carried out as in the materials and methods. SMARTpools causing significant loss of viability effects (where Z≤−3) are shown in bold.
(0.14 MB XLS)

Table S2 Title of dataset: Combined RNAi and expression data from five cancer cell lines Description of dataset: Z scores are shown for SMARTpools for all 779 gene. For each of the corresponding genes, transcript expression data is shown, generated by Illumina profiling [15]. In this case, average signal from each Illumina probe is shown. Significance of correlation between expression and siRNA Z score with Pearson correlation coefficient and Spearmans rank correlation coefficient.
(0.40 MB XLS)

Table S3 Title of dataset: Array Comparative Genomic Hybridisation (aCGH) data from five cancer cell lines Description of dataset: Genome-wide aCGH profiling was performed as described in the materials and methods. Average signal from each BAC is shown.
(8.95 MB XLS)

Figure S1 Correlation between gene copy number and Z score. Scatter plots illustrating the relationship between gene copy number and sensitivity to siRNA. Horizontal dashed lines represent the threshold for copy number gains (aws ratios>0.12) and vertical dashed lines represent the threshold for significant loss of viability effects.
(0.10 MB TIF)

Figure S2 Gene silencing of WEE1. a. Cells were transfected with SMARTPool siRNA or one of the component siRNAs from each SMARTPool as shown. Forty-eight hours after transfection, RNA was extracted and quantitative real-time PCR performed. Specific gene expression in each sample was normalised to that of a house keeping gene (GAPDH) and standardised according to gene expression in cells transfected with a control, non-targeting siRNA (siCONTROL). Each bar represents data from triplicate transfections, with error bars representing SEM. * = p<0.05 vs siCONTOL, Student's t test. B. Multiple WEE1 siRNAs cause selective killing of CAL51 cells, when compared to MCF7 cells. Cells were transfected with SMARTPool siRNA or one of the component siRNAs from each SMARTPool as shown. Cell viability measurements were performed and surviving fractions calculated as in the materials and methods. Each bar represents data from triplicate transfections, with error bars representing SEM. * represents significant (p<0.05) loss of viability in CAL51 cells vs MCF cells transfected with the same siRNA.
(0.08 MB TIF)
