# Supplementary figures and images for: Integrated Functional, Gene Expression and Genomic Analysis for the Identification of Cancer Targets
Source: PLoS One. 2009 Apr 9;4(4):e5120. doi: 10.1371/journal.pone.0005120 (PMC2663812; doi:10.1371/journal.pone.0005120)

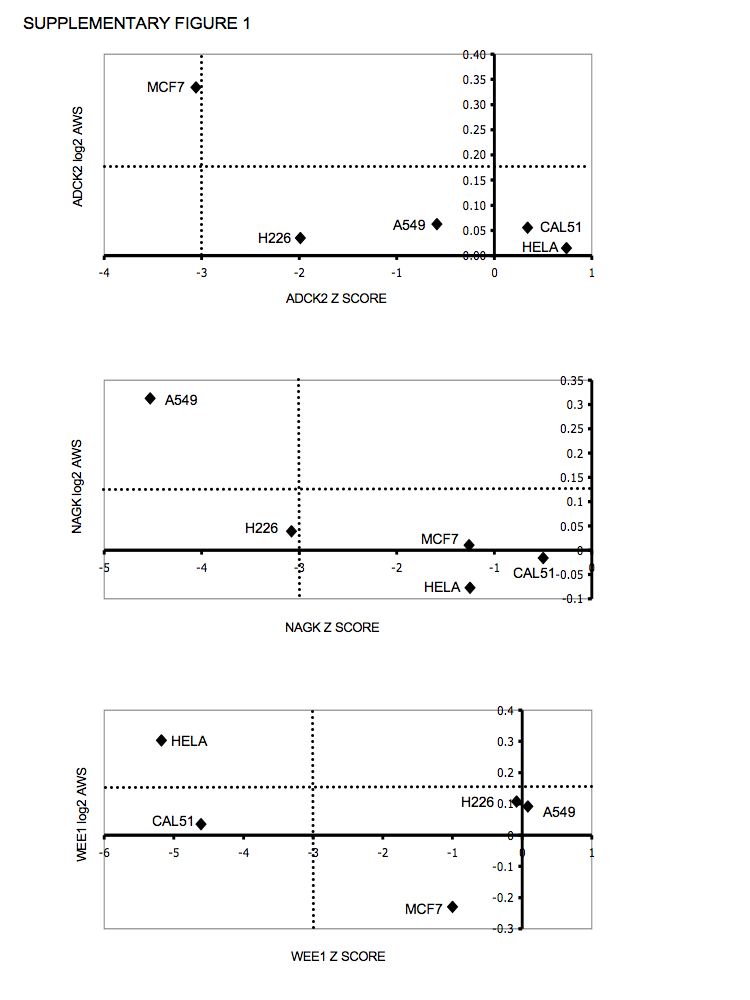

Supplement: Figure S1 — Correlation between gene copy number and Z score. Scatter plots illustrating the relationship between gene copy number and sensitivity to siRNA. Horizontal dashed lines represent the threshold for copy number gains (aws ratios>0.12) and vertical dashed lines represent the threshold for significant loss of viability effects. (0.10 MB TIF) [file pone.0005120.s004.tif]

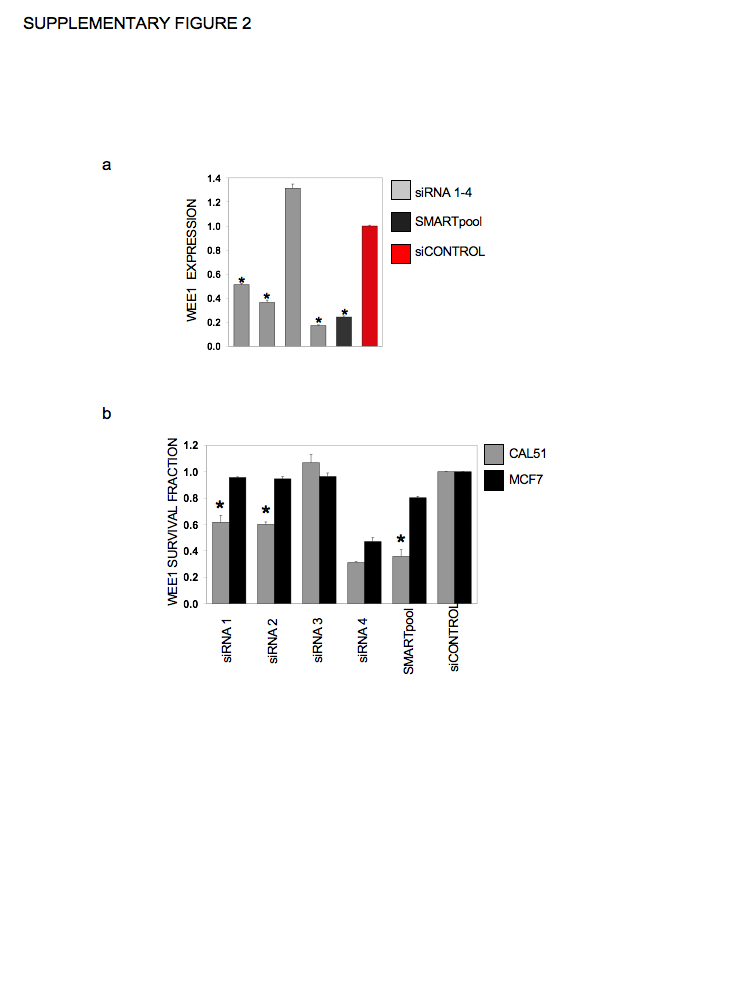

Supplement: Figure S2 — Gene silencing of WEE1. a. Cells were transfected with SMARTPool siRNA or one of the component siRNAs from each SMARTPool as shown. Forty-eight hours after transfection, RNA was extracted and quantitative real-time PCR performed. Specific gene expression in each sample was normalised to that of a house keeping gene (GAPDH) and standardised according to gene expression in cells transfected with a control, non-targeting siRNA (siCONTROL). Each bar represents data from triplicate transfections, with error bars representing SEM. * = p<0.05 vs siCONTOL, Student's t test. B. Multiple WEE1 siRNAs cause selective killing of CAL51 cells, when compared to MCF7 cells. Cells were transfected with SMARTPool siRNA or one of the component siRNAs from each SMARTPool as shown. Cell viability measurements were performed and surviving fractions calculated as in the materials and methods. Each bar represents data from triplicate transfections, with error bars representing SEM. * represents significant (p<0.05) loss of viability in CAL51 cells vs MCF cells transfected with the same siRNA. (0.08 MB TIF) [file pone.0005120.s005.tif]
